# Imaging junctions in two-dimensional semiconductor nanosheet networks

**DOI:** 10.1038/s41699-025-00609-6

**Published:** 2025-10-31

**Authors:** Jelena Pešić, Simon Leitner, Joseph Neilson, Igor Stanković, Muhammad Zubair Khan, Dragana Tizić Matković, Adam G. Kelly, Tian Carey, Jonathan Coleman, Aleksandar Matković

**Affiliations:** 1 Chair of Physics, Department Physics, Mechanics and Electrical Engineering, Montanuniversität Leoben, Leoben, Austria; 2 Laboratory for 2D Materials, Center for Solid State Physics and New Materials, Institute of Physics Belgrade, University of Belgrade, Belgrade, Serbia; 3 School of Physics, Trinity College Dublin, Dublin, Ireland; 4 Scientific Computing Laboratory, Center for the Study of Complex Systems, Institute of Physics Belgrade, University of Belgrade, Belgrade, Serbia; 5 Chair of Resource Mineralogy, Montanuniversität Leoben, Leoben, Austria; 6 I3N/CENIMAT, Faculty of Science and Technology, Universidade NOVA de Lisboa, Campus de Caparica, Caparica, Portugal

**Keywords:** Nanoscale materials, Two-dimensional materials

## Abstract

This study explores the challenges associated with translating electrical characteristics of individual two-dimensional semiconductor nanosheets into a network of partially overlapping sheets. Such systems typically suffer from high-energy barriers required to overcome the junctions formed between the adjacent nanosheets, and consequently quench the current passing through the network. We use *in-operando* Kelvin probe force microscopy to image electrostatic potential profiles during the operation of MoS_2_ nanosheet network transistors. Direct imaging of the potential drops allows us to distinguish contributions from individual nanosheets and those from junctions, correlated by the junction-related potential drops with the network morphology. A diagram-based model is developed to describe the system numerically and to estimate the current path formation probabilities. Finally, a correlation with the integral electrical characteristics of the nanosheet-based transistors is made using a robust Y-function approach. It is shown that the total junction resistance is well estimated by the proposed equivalent circiut model.

## Introduction

Extensive research has been devoted to explore the potential of solution-processed 2D nanosheets networks across various electronic applications^1–3^. These materials offer promising physicochemical properties for low-cost and scalable applications in optoelectronics, photocatalysis, sensing, and photovoltaics^1–6^. The motivation lies in harnessing the inherent properties of individual nanosheets and their potential for assembly into large-area surfaces^7,8^.

However, in the context of semiconductor nano-networks, these expectations often go unmet^3,8,9^. Challenges arise in translating the exceptional characteristics of individual nanosheets into large-area functional surfaces, primarily due to the formation of junctions between nanosheets^1,3,9–12^. The “bottlenecks” for the current flow in these systems are high energy barriers at the junctions formed between adjacent nanosheets^2,3,13^. These energy barriers impede electron flow, leading to a substantial reduction in the overall current passing through the network^2,3,14^. Understanding junctions and their influence on the conduction behavior of nanonetworks^3,8^ is essential for further development of solution-processed semiconducting materials, and their integration into thin film transistors (TFTs), sensors, and other electronic devices^15–20^.

This study correlates the microscopic and macroscopic electrical properties of predominantly monolayer MoS_2_, as an exemplary 2D semiconducting nanosheet network system. Supplementary Figs. S1–3, present correlated AFM, Raman, photoluminescence, and scanning-electron-microscopy data for representative MoS_2_ flakes, collectively confirming that the film is composed predominantly of monolayers. To directly image the microscopic distribution of the potential drops across the channels in the TFTs, we employ *in-operando* frequency-modulated Kelvin Probe Force Microscopy (FM-KPFM), offering ~ 10 nm spatial resolution of the potential drops in the nanosheet network during device operation^21–25^.

We further establish a robust model to estimate the probability of the formation of current paths between the source (S) and the drain (D). We correlate our microscopic investigations of the junctions with integral macroscopic electrical measurements^9,26,27^. Y-function-based fitting of the *I*_D_(*V*_G_) curves enables estimating a sum of all non-field modulating potential drops, providing excellent agreement with the junction-related potential drops extracted from the KPFM. Although the presence of potential drops at junctions in 2D nanosheet networks is anticipated, our study uniquely quantifies these drops with nanometer-scale resolution using *in-operando* KPFM. This direct imaging, combined with a robust Y-function approach, bridges the gap between microscopic potential variations and macroscopic electrical characteristics, offering a comprehensive framework for understanding current path formation. A key objective of this work is to link macroscopic modelling of parasitic elements with nanoscale imaging of potential drops. To enable a meaningful comparison, we adopted a meandering, wide-channel geometry, which maintains high on-state currents and preserves realistic device performance. Since the width of the channels is much larger than their length, we expect several hundred conduction paths to form in each device during integral electrical measurements. To obtain a statistically relevant set of local measurements, we measured over 100 KPFM maps of the same devices used in the macroscopic tests.

## Results

### Formation and statistics of conduction paths

**Fig. 1:**
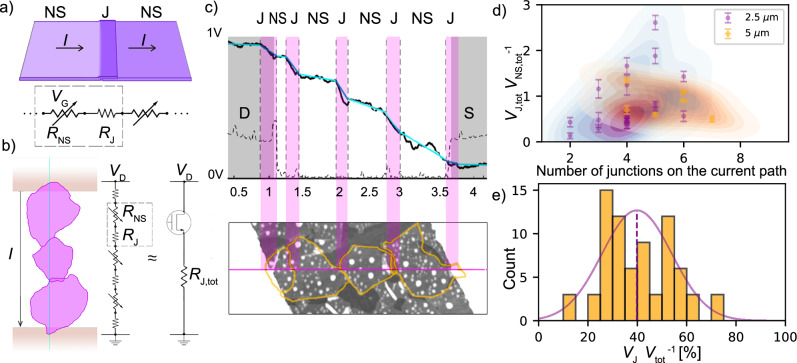
Impact of Junctions on Current Paths in Nanosheet TFTs. **a** Schematic illustrating a junction consisting of the two overlapping nanosheets. We can approximate this with 2 resistances of individual nanosheets (NS) that are gate dependant (*V*_*G*_) and a resistance of presumably non-gate dependant junctions (J). **b** An illustration of a single current conducting path thought the network, consisting of a linear array of nanosheets and junctions between them. This network can be treated as a resistor series consisting of nanosheets, with resistance *R*_NS_, and junctions with resistance *R*_J_. An equivalent two component circuit, consisting of an n-type TFT with resistivity *R*_NS,tot_(*V*_G_) and *R*_J,tot_ is presented on the right side of the panel. **c** Measured potential drop profile along the channel between source (S) and drain (D) during device operation, with marked NS and J regions. Junction areas are highlighted in lilac in both the electrical field potential lines and the topography map. Black dashed line indicate the topography profile. Lower panel: Topography map around the selected current path, marked with a violet line. **d** Correlation between normalized voltage drops on nano-sheets and junctions, relative to the number of junctions observed on each current path. Purple circle markers are for 2.5 μm transistors and orange diamond for 5 μm ones. Representative data clouds are extrapolated using Kernel Density Estimation. **e** Statistics for the percentage of voltage drop across the junctions *V*_J_ relative to the total voltage *V*_tot_ drop along the current path. Solid purple line represents a Gaussian fit.

Figure 1a illustrates a junction formed by two overlapping nanosheets, each individually exhibiting gate-dependent resistance^3,21^. The junction resistance in the MoS_2_ nanosheet networks is simplified to be independent of gate voltage. This approximation is discussed in more detail later in the text. Each nanosheet-junction pair along the current path can be modeled as these two resistances connected in series, repeating on the path. For any given current path (Fig. 1b), we assume that carriers move through a sequence of nanosheets, crossing inter-sheet junctions. Consequently, an individual current path can be represented as a linear array of nanosheet-junction pairs. On each current path its sequence of voltage drops is recorded by KPFM. Total current path between drain and source electrodes can be expressed as two equivalent components, *n*-type TFT that represents all nanosheets and exibits gate-dependent resistance of *R*_NS,tot_(*V*_G_), and the total junction resistance contribution *R*_J,tot_.

We expect conductive paths to form between the electrodes, following the shortest route that crosses the sheets while bypassing non-covered areas in the film. Topography and contact potential difference (CPD) voltage maps were recorded across randomly selected areas of the transistors. In almost all images – considering 2.5 μm and 5 μm long channels – at least one path with continuously overlapping sheets was observed, and assigned to the conduction path. All measured CPD profiles are normalized and corrected for the work function differences (details are provided in SI Sections 1.2 and 1.3).

In the topographic analysis, individual MoS_2_ nanosheets are distinctly identifiable. The height profiles indicate that almost all sheets are monolayer MoS_2_. Topography characteristics correlate to CPD profiles allowing to assign the contribution of the individual sheets and junctions, parameterized with linear fits (Fig. 1c). We have extracted and fitted numerous current paths considering multiple transistors with 2.5 μm (purple) and 5 μm (orange) channel length. A ratio between the total potential drop along the conductive path associated with the junctions, (*V*_J,tot_), versus the total potential drop associated with the nanosheets, (*V*_NS,tot_), is presented as a function of the TFT’s channel length in Fig. 1d. We can conclude that, irrespective of the channel length, a relative ratio of the potential drops between the sheets and the junctions remains rather uniform. Consequently, we anticipate that current paths frequently form across the entire width of the transistor. Figure 1e illustrates an average percentage of voltage drop across the junctions relative to the total voltage drop along the current path for all studied current paths. For the examined system, approximately 30–50% of the potential drop is associated with the junctions, indicating that nearly half of the voltage from source to drain is lost due to junction resistance. This quantitative range applies specifically to networks of liquid-phase-exfoliated MoS_2_ monolayers with an average radius of 0.3 μm, deposited onto SiO_2_/Si substrates under the processing conditions detailed in the Methods section. However, the size-dependent analysis demonstrates that even micron-scale MoS_2_ networks remain junction-limited, with R_*J*_ reaching giga-ohm values and exceeding R_*NS*_^3^. Complementary temperature-dependent impedance and nanoscale potential-mapping studies^3^ reveal phonon-limited, band-like transport within basal planes but thermally activated hopping across interfaces, again giving R_*J*_ > R_*NS*_ in thicker, higher-mobility MoS_2_ networks^3,28^. Collectively, these reports indicate that, unless interfaces are deliberately engineered, junction resistance continues to dominate transport irrespective of flake size.

### Diagram-based network model and validation

**Fig. 2:**
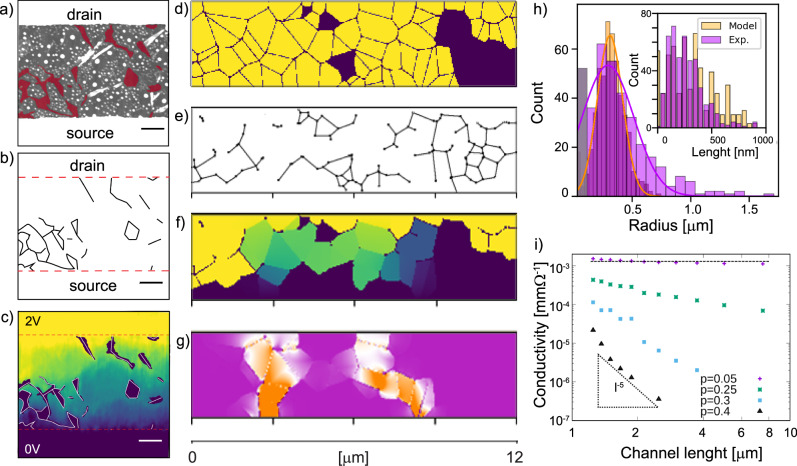
Topography-Informed Modeling of Conductivity in Nanosheet Networks. **a** Topography of a 2.5 μm TFT (scale-bar 500 nm, *z*-scale 8 nm). A red mask highlights the non-covered parts of the channel active area, with the edges of the source (S) and drain (D) with dashed red lines. **b** Distribution of non-conductive edges extracted from topography. **c** Electrical potential drop map of the same area as the topography, with regions identified as holes in the topography removed from the potential map. Recorded during device bias of +2 V, yielding *I*_D_ ≈ 3 × 10^−9^ *A* × μm^−1^. **d** Representative spanning cluster of the model for the generated nanosheets over an equivalent 3 × 12 μm^2^ area. The spanning cluster corresponds to the topography of the TFT. Yellow denotes connected nanosheets, while dark violet indicates electrostatic holes in the film. **e** Corresponding network of non-conductive edges from the model-generated topography. **f** Simulated electric potential drop map, and **g** the corresponding current density map. **h** Experimental and model average sheet radius with Gaussian fits. Inset: Statistics of non-conductive edge length in both the model and experiment. Orange markers correspond to model and violet to experimental data. **i** The model parameter describing conductivity of polygon edges (*p*) was varied to simulate the different quality of contact between nanosheets and to observe dependence of conductivity between electrodes on the channel length (*l*), presented in semi-log scale. If many edges of the generated polygons are conductive *p* = 0.05 (corresponding to well connected network of nanosheets) there is negligible dependence on channel length. Conversely, when there are many nonconductive edges, i.e., few conduct *p* = 0.4 (which corresponds to poorly connected network of nanosheets), the dependence on length of the channel is significantly higher.

Figure 2 compares the experimental data from KPFM with a representative example of the model system. Figure 2a shows AFM topography with marked non-covered areas of film by a red mask. Edges of the non-covered areas are treated as non-conductive ones and we can extract this as a network of experimentally observed non-conductive edges in the system (Fig. 2b). Regions marked as holes in topography are removed from the potential image (Fig. 2c). From examination of correlated topographical and potential drop profiles of 20 distinct devices and the analysis of roughly 100 current paths, we develop a diagrams-based model to simulate the networks of conductive sheets with interconnecting junctions. The model defines geometrical regions, which we refer to as cells, that correlate to the nanosheets. These are constructed such that any point within a given cell is closer to its seed (generated using a uniform random distribution) than to any other seed. The final system describes a densely packed network of polygonal sheets and their contacting edges. The probability of two touching edges to conduct increases with the inverse square of their length. This is justified by the fact that contact forms between two edges, and the probability of no conduction depends on the length of each edge on both sides. To evaluate electrical properties, we applied Kirchhoff’s laws to the network, discretizing the system and assigning conductivity to each discrete square surface region (pixel) based on its position to ensure current balance at every node. For more details, please refer to the SI Section 1.6.

Figure 2d shows the spanning cluster of the model system i.e., the simulated random distributions of the sheets densely packed between the two electrodes. The darker colored sheets represent the ones without any electrical connection to the surrounding, and correspond to the non-covered areas of the film. Figure 2e depicts the distribution of the non-conductive edges. These non-conductive edges interrupt potential current paths and affect the network’s conductivity. Figure 2f illustrates the resultant, calculated potential drops across the network^29^. For presentation in this paper, we constructed a model system with a channel length of 3 μm and a width of 12 μm. The model can capture many other relative sizes between nanosheets and device geometry factors by adjusting the appropriate seed density and length/width ratios (SI section 1.6 and 1.7; Fig. S10–13) Fig. 2g shows model’s prediction of the current paths through the system, as normalized intensity of the current density. Current flow is from the top edge (biased) to the bottom edge (grounded), that act as the transistor’s electrodes. From the model, we estimate the density and the complexity of the dominant current path formation, and correlate these with the distribution of conductive and non-conductive edges. These dominant current paths are often referred to as ’winner-takes-all’ paths, a phenomenon discussed in the literature^30,31^. To address this, further work on film formation is required to develop more uniform films with improved spatial current distribution, ensuring a more balanced and efficient flow of current across the material.

To validate that our system accurately reflects the experimental data, we have made a comparison based on geometric parameters of the nanosheets and their representative cells. Figure 2h presents a sheet radius distribution comparison between the AFM data^32^, and partitioning of the model, with an average radius of 0.3 μm in both datasets. It is worth noting that due to the experimental method limitations, sheets smaller than 20 nm cannot be clearly resolved.

Further, we compare the length distribution of non-conductive edges in both the experimental data and the model, showing a good match (Fig. 2h inset). With our assumption that the probability of two touching edges being non-conductive depends on the inverse square of their length, we matched the experimental distribution of non-conductive edges. This probabilistic approach accurately reflects the variability and randomness observed in the actual conductive networks.

Our model captures as well a dependence of conductivity on channel length (Fig. 2i). By varying the model parameter that describes the quality of contact between nanosheets, we observed changes in conductivity relative to channel length. In a perfectly connected network, the conductivity is independent of the channel length. For networks with breaks and junctions between the adjacent NSs, indicated by fewer connected polygons in our model, the dependence of conductivity on channel length is much steeper. Previous studies on networks have shown relationships such as $R \sim L\times {(L/{L}_{0})}^{{\rm{n}}}$, where *L*_0_ is a characteristic length and *n* increases from 0 with increasing disorder^33^. Similar effects are observed with impedance, where the non-linearity in *L* can be directly attributed to the junctions^34^. Further, it was reported that better-connected networks exhibiting less pronounced increases in resistance as channel length grows^35^. This observation supports the notion that longer channels increase the likelihood of encountering current bottlenecks^3^. In our system, disorder arises from factors such as overlap length variation, sheet misalignment, and non-conductive edges. By fitting our averaged experimental data, we estimate the scaling exponent to be *n* ≈ 0.4, placing the system well above the percolation threshold. The model captures this through the probabilistic formation of junctions, where the conduction probability scales inversely with the overlap length, aligning with experimental observations.

Considering the results of the model, on average 25% of the total device channel width is contributing to the current flow from source to drain. This supports the assertion that the current follows the path of least resistance, as evidenced by conductive AFM measurements on electrochemically exfoliated MoS_2_ networks produced via inkjet printing^36^. Scaling up the model shows this behavior consistently across the all tested widths and lengths. Our model indicates that even a modest proportion, such as 15–20%, of non-conductive edges and below 20% of holes in the film can lead to a substantial decrease in the probability for the conductive paths to form in the network. However, current paths do frequently form, the flow of current remains prevalent, and the transport is likely not defined by only a small number of hot spots.

### Junction analysis

To delve deeper into the junction formation between nanosheets and their impact on the overall current flow, we focus on the adjacent nanosheet overlap. The simplistic view of two monolayer sheets overlapping, suggesting formation of a homojunction and enhanced conductivity in the overlap region. However, complexities arise from nanosheet production and deposition processes. These often lead to unexpected, less desirable, and less predictable behavior in the overlap region.

**Fig. 3:**
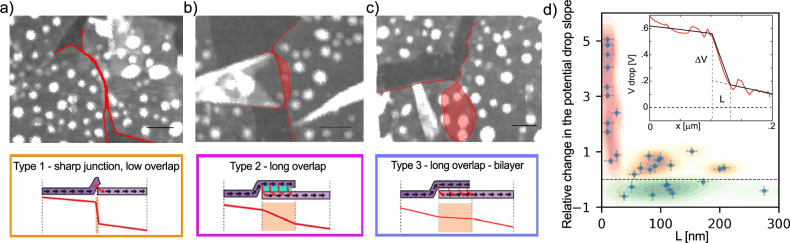
Correlation Between Overlap Geometry and Junction Resistivity in Nanosheet Networks. **a** An example of a sharp junction between sheets with minimal overlap resulting in poor contact. Scale-bar in **a**–**c** is 100 nm, and *z*-scale is 10 nm. **b** The long overlap between sheets, coupled with trapped impurities between two monolayers, forms junctions that increase resistivity compared to *R*_NS_. **c** Long overlap between sheets, pushing out residue and making good lateral contact between nano-sheets. This kind of junctions are beneficial for conduction as it reduces resistivity on the path. **d**) Relative change of the potential drop slope between the junction and the adjacent sheets per unit length of overlap in junction and as a function of the junction overlap length. Representative data clouds extrapolated using Kernel Density Estimation. Inset: Schematic of potential drop analysis of Type 1 junctions.

In Fig. 3a–c, we distinguish three typical examples of junctions that may form between two sheets. Further details are provided in SI Sections 1.3 and Fig. S5. Figure 3a depicts what is labeled in this work as a type 1 junction, and captures minimal overlap between the sheets. This type of contact is characterized by a sharp drop in voltage. From our measurements, we conclude that such junctions typically have relatively small overlap lengths, up to 50 nm.

In Fig. 3b, we present a typical junction characterized by a long overlap with trapped impurities, labeled as type 2 junction. These junctions typically exhibit overlap regions ranging from 50 to 200 nm. Due to the presence of trapped impurities, it is not appropriate to consider the overlap region as potentially formed bilayer. In contrast to sharp junctions with minimal overlap, the change in slope of the linear fit between the junction and the flakes is not significantly pronounced in this case.

The third type of junctions can be viewed as constructive in terms of the current flow, labeled as type 3 junctions (Fig. 3c). These junctions feature long overlaps where residues from synthesis and deposition processes are effectively pushed out, allowing the sheets to form good contact between them. We observe a smaller slope in potential than that on individual sheets. This suggests a reduction in resistance compared to the sheets that form the junction. Given the solution-processed fabrication and absence of any crosslinking/sintering chemistry, the overlaps between MoS_2_ monolayers constitute van der Waals interfaces. Their electrical coupling is therefore dictated by overlap geometry, twist, and interfacial cleanliness: trapped residues act as nm-scale spacers that raise the effective tunnelling/hopping barrier, whereas residue-free extended contact approaches the behavior of a local bilayer, reducing the local drop. The inter-flake residue is composed mostly of polyvinylpyrrolidone (PVP) from the ink, accompanied by traces of solvent, adventitious organics, and adsorbed water. A multifaceted cleaning and processing strategy–described in more detail in the Methods section–could potentially enhance network connectivity.

The collective contribution of these junctions, encompassing any of the three types we have identified, significantly influences the observed potential drop along the path. From the datasets of the experimentally observed current paths, we summarize the statistics of the potential slope change at the junctions per length of the NS overlap, as: *ΔV*/*L*_J_ = d(*V*_J_)/d*x* − d(*V*_NS_)/d*x*. Here, *L*_J_ refers to the length of the nanosheet overlap, considered to be in the direction of the current flow *x*, and should be distinguished from the width of the overlap region between the nanosheets. We assume that the two sheets forming a junction possess similar resistance, and that an equal current flows on both sides of the junction.

In the analysis presented in Fig. 3d, we treat each junction as independent, observing the relative change of voltage on the junction in relation to its length (an example in Fig. 3d inset). In Fig. 3d, we define three distinct areas. The orange area represents low-overlap junctions of type 1, characterized by high resistance. NS overlaps smaller than 10 nm could not be identified in topographical images due to the AFM resolution limits, but they are detected in CPD measurements; thus, we classify them as 9.999 nm to maintain a consistent comparison. For larger overlaps, with lateral sizes exceeding 50 nm, we no longer observe type 1 junctions, but rather statistically equally distributed type 2 and type 3 junctions. We can conclude that more than 50 nm overlap between NSs is needed to have less disruptive impact of junctions on potential drops. The junctions beneficial for current flow (type 3) are denoted in blue and have negative values of *ΔV*/*L*_J_. Type 2 junctions with resistances higher than individual sheets are represented in purple. A value of *ΔV*/*L*_J_ = 0 would imply an equal resistance between the sheets and junctions, resulting in a constant slope of the voltage drop line. Bubbles, trapped impurities, and residue from the deposition process can act as separators between the nanosheets in the overlapping region, effectively increasing the resistance of the junction.

### Device-level transport and Y-function extraction

**Fig. 4:**
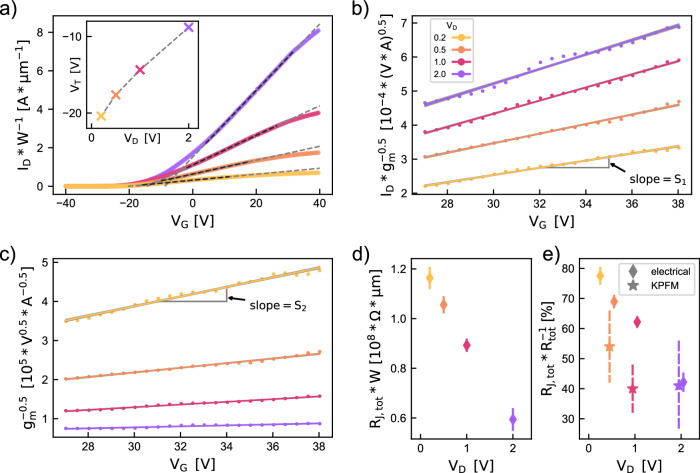
Transfer Curve Analysis and Junction Resistance Mapping. **a** Transfer curves taken at different *V*_D_, and at 300 K. *V*_T_ is determined from linear fits (dashed lines, black dashed area used for fitting), and *V*_T_(*V*_D_) dependence is presented in the inset of **a**). Dashed lines in the inset are guides to the eye. **b** and **c** respectively show fitting linear functions to ${I}_{{\rm{D}}}/\sqrt{{g}_{{\rm{m}}}}$ and $1/\sqrt{{g}_{{\rm{m}}}}$, in the strong inversion regime (*V*_G_ > 27.5 *V*). Barely visible shading denotes the 1*σ* confidence band for the fit parameters. The slopes of the fit functions are the *S*_1_ and *S*_2_ parameters, respectively. **d** Gate-independent width scaled junction resistance *W* × *R*_J,tot_ as a function of *V*_D_. Error bars show 1*σ* confidence interval. **e** Ratio of *R*_J,tot_ to the total resistance of the device as a function of *V*_D_, with 1*σ* error bars. Stars represent KPFM data and diamonds are electrical measurements. The color legend in (**b**) is valid for all of this figure.

Lastly, we correlate our microscopic investigations of the junctions in semiconducting NS networks with commonly applied macroscopic electrical measurements. Figure 4 provides a detailed analysis of electrical measurements and device modeling for a nanosheet transistor with a 5 μm channel length, 10,000 μm channel width, and 85 nm thick SiO_2_ together with a highly doped Si chip as a global back-gate. A wide, meandering electrode design was chosen specifically to deliver high drain currents and preserve optimal transistor performance in this study. Figure 4a presents a typical series of the electrical transfer curves of a device with varied drain voltage (*V*_D_), recorded at 300 K and at a constant sweeping speed of 5 V s^−1^. The drain-source currents (*I*_D_) are scaled with the device width, and also adjusted by the fraction of the area estimated to be active (25%) according to the prediction of the current path density obtained from our model. Linear fits to the strong inversion region are denoted by dashed gray lines. The linear threshold voltage (*V*_T_) was extracted, and shows a shift to more positive values with increasing *V*_D_. ON-OFF ratios were estimated to be between 10^3^ and 10^4^ (see also SI Section 1.3 and Fig. S7).

We utilize a fitting approach of the *I*_D_(*V*_G_) curves employing a Y-function-based model^37^. This model is used to analyze contact resistance contribution in TFTs^38,39^ (further details are provided in SI Sections 1.5). We assume that the the contact resistance parameter of the model corresponds to a sum of all non-field modulating resistance elements, denoted as *R*_J,tot_. Obtained total junction resistance values are compared to the potential drops from individual current paths observed by KPFM measurements. We approximate any current path as comprising linear and non-linear components, corresponding to a sum of gate-dependant NS resistances and junction resistances (Fig. 1b).

The resistance of the metal contacts and potential Schottky barrier formation at the 2D semiconductor/metal interface also influence the overall device characteristics^21,22^. In-operando KPFM allows us to distinguish the voltage drop at nanosheet junctions from that at the metal contacts. Analysis of the potential profiles shows that no more than 10 ± 2% of the junction-related drop originates at the Au/MoS_2_ interfaces; the remainder occurs at MoS_2_/MoS_2_ overlaps (Fig. S6). Because both NS-NS junctions and the NS themselves are highly resistive, contact resistance is not the dominant source of bias loss in this network, unlike in many single-crystalline 2D devices^21,22^. Accordingly, the Y-function analysis treats contact resistance as part of the total non-field-modulating term, and the junction resistance reported from both KPFM and Y-function fitting is a single lumped value that combines Au/MoS_2_ and MoS_2_/MoS_2_ contributions. The potential drop associated with the Au/MoS_2_ interface is localised almost entirely at the grounded source electrode, indicating an electron-injection barrier characteristic of a Schottky contact.

Y-function fitting^40^ was done according to the method described by Jain et al.^37^, following an equation for a series of a long-channel ideal transistor in the linear regime and a resistor *R*_J,tot_. The transconductance of the device *g*_m_ = ∂*I*_*D*_/∂*V*_*G*_ can be expressed as following:

1 $$1/\sqrt{{g}_{{\rm{m}}}}={\left(\frac{L}{{\mu }_{0}{C}_{{\rm{G}}}{V}_{{\rm{D}}}W}\right)}^{1/2}[1+\theta ({V}_{{\rm{G}}}-{V}_{{\rm{T}}}-{V}_{{\rm{D}}}/2)]$$

where $\theta \,=\,{R}_{{\rm{J}},{\rm{tot}}}{\mu }_{0}{C}_{{\rm{G}}}\frac{W}{L}$, *C*_G_ is the gate capacitance per unit area, *V*_T_ is threshold voltage and *μ*_0_ is intrinsic mobility. Further, multiplying the $1/\sqrt{{g}_{{\rm{m}}}}$ function with *I*_D_ that is expressed as a series of an linear-regime transistor and *R*_J,tot_ resistor, leads to the expression for the *Y* function^37,40^:

2 $$Y\equiv \frac{{I}_{{\rm{D}}}}{\sqrt{{g}_{{\rm{m}}}}}={\left({\mu }_{0}{C}_{G}{V}_{{\rm{D}}}\frac{W}{L}\right)}^{1/2}({V}_{{\rm{G}}}-{V}_{{\rm{T}}}-{V}_{{\rm{D}}}/2)$$

By fitting linear functions to ${I}_{{\rm{D}}}/\sqrt{{g}_{{\rm{m}}}}$ and $1/\sqrt{{g}_{{\rm{m}}}}$ in the strong inversion regime (*V*_G_ > 30 *V*) we extract the parameters *S*_1_ and *S*_2_ obtained as the slopes from Fig. 4b,c. *R*_J,tot_ can then be calculated as *R*_J,tot_ = (*S*_2_ ⋅ *V*_D_)/(*S*_1_). The data with linear fits are provided in Fig. 4b,c, with a background shading showing the 1*σ* confidence band for the fit parameters. The calculated *R*_J,tot_ values are shown in Fig. 4d as a function of the applied bias. The junction resistance R_*J*_ is inherently bias-dependent. Temperature-dependent impedance spectroscopy on comparable MoS_2_ networks shows that inter-sheet transport follows Miller-Abrahams hopping between 150 K and 300 K, whereas intraflake transport remains phonon-limited and band-like, this dual mechanism accounts for R_*J*_ > R_*NS*_ under most bias conditions^6^. A gate-dependent study reported an activation energy of ≈ 55 meV at T ≳ 220 K that decreases with positive V_*G*_, implying a reduction of R_*J*_ in the on-state^6^. We treat R_*J*,*tot*_ as gate-independent for the Y-function fit, an expedient first-order approximation that reproduces the device characteristics and aligns with the KPFM statistics, while explicitly noting that a modest V_*G*_ dependence is expected. To probe this further, we include in the Supplementary Information a temperature series of transfer curves analyzed via a standard Schottky-barrier-height extraction^41–43^, the resulting barrier heights (SI Fig. S8) corroborate a gradual lowering of the electron-injection barrier with increasing gate bias, while the accompanying output curves (SI Fig. S9) display a pronounced S-shaped bend at low V_*DS*_, consistent with significant contact and junction resistance.

The proportion of the junction resistance to the total resistance of the device is shown in Fig. 4e, with error bars denoting 1*σ* confidence intervals. With diamonds, electrical measurement data is presented and stars denote KPFM measurements. Nanoscale KPFM line scans measure the voltage drop at each MoS_2_-MoS_2_ and Au-MoS_2_ interface, whereas the Y-function fit yields a single non-field-modulating resistance for the whole device. When the individual KPFM drops are summed, the resulting junction resistance agrees, within experimental uncertainty, with the Y-function value. This consistency suggests that the local KPFM maps and the device-level analysis capture the same dominant resistive elements. In comparison to KPFM-extracted data, the integral Y-function-based model yields higher estimate of the total junction resistance. The Y-function-based model considers the response of the entire device, and all of its conduction paths, while KPFM-extracted junction contribution is somewhat dependent on the selection of the current paths, which is a likely source of the observed discrepancy. The introduced approximation of the Y-function model allows to separate the junction from the sheet contributions, and enables to adopt the method to estimate the quality of the junctions in semiconducting nanosheet networks from electrical transfer curves.

## Discussion

This study offers an insight into how complex morphological characteristics of 2D semiconducting nanosheet network TFTs influence their electrical behavior. Our combined approach consists of local KPFM and integral electrical measurements, device and network modeling. We compare locally observed potential drops from individual current paths with the macroscopic response of the TFTs.

Through imaging of the potential drop profiles and numerical modeling of the artificially generated NS networks, we conclude that discreet current flow paths form rather uniformly across the entire device. The results indicate that conductive routes can twist between non-conductive areas, maintaining the current flow meandering around non-conductive edges and holes in the film. Within these current paths, still, significant voltage drops are observed at the junctions between the nanosheets. We further illustrate the importance of the NS-NS overlap length on the resistivity of the junctions, underscoring the critical role of further development of the nanosheet deposition techniques.

Lastly, we have demonstrated a correlation between local potential drops observed by KPFM with integral electrical measurements and propose the use of a robust Y-function fitting method to extract the contribution of the junctions. The impact of the junctions in the total device performance can be rather well captured by approximating the entire network as a serial connection of a perfect transistor depicting the sum of the NSs and a gate-independent resistor accounting for the sum of all junction contributions.

## Methods

### Preparation of MoS_2_ monolayer ink

MoS_2_ monolayer enriched dispersions were prepared by the electrochemical exfoliation of molybdenite crystal (natural origin, Krupka, Czech Republic). A two-electrode electrochemical cell was set up with a ~ 1 mm thick, 0.5 cm^2^ cleaved piece of MoS_2_ crystal as the cathode, and ~ 1 mm thick, 1 × 6 cm strip of graphite foil (Alfa Aesar) as the anode. In a 50 mL beaker, the electrodes were submerged in 40 mL of 12.5 mg (mL)^−1^ solution of tetraheptylammonium bromide [THA]+[Br]- (Sigma Aldrich) in acetonitrile (≥99.5%, Sigma Aldrich). A potential of 7 V was applied across the electrodes for 1 h. After the MoS_2_ crystal, which is expanded due to intercalation of [THA]+, was rinsed with acetone (≥99.5%, Sigma Aldrich) three times to remove excess bromine. Then, the expanded MoS_2_ crystal was added to 80 mL of 2% w/v solution of polyvinyl pyrrolidinone (PVP) (MW: 40,000, Sigma Aldrich) in dimethylformamide (Sigma Aldrich) and bath sonicated for 1 h. The dark green dispersion of MoS_2_ was first centrifuged at 958 RCF for 30 min to separate the sediment of bulky nanosheets and unexfoliated MoS_2_. The supernatant was collected and subject to another centrifugation of 3830 RCF for 60 minutes and the sediment containing monolayer enriched nanosheets was collected in 80 mL of isopropyl alcohol (IPA) (≥99.5%, Sigma Aldrich) for further processing. The IPA dispersion was washed to remove excess PVP by another round of centrifugation at 3830 RCF for 60 min followed by redispersion in 80 mL of IPA to create the final MoS_2_ monolayer ink.

### Liquid interface deposition of MoS_2_ films

**Fig. 5:**
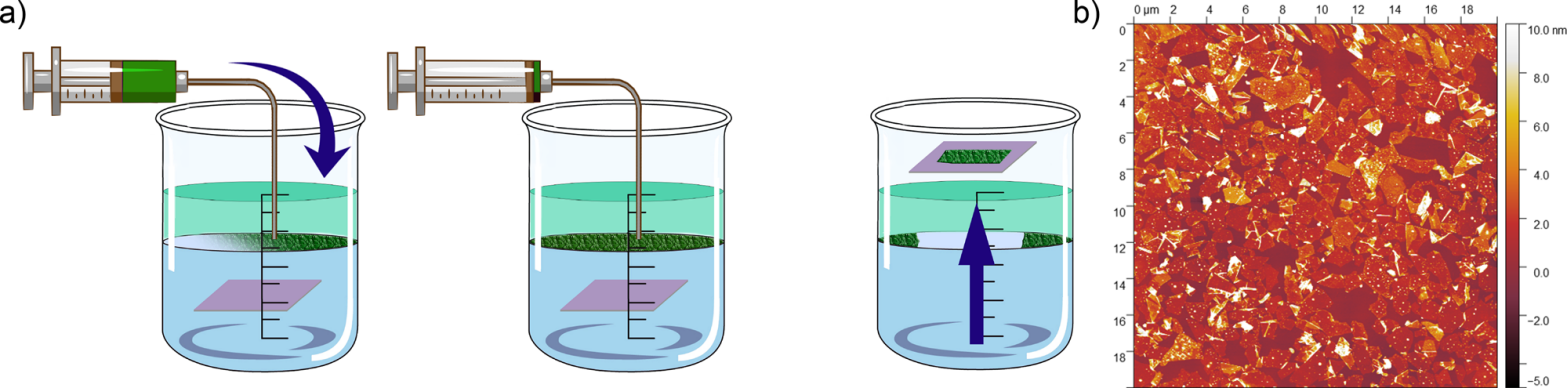
MoS_2_ Film Deposition via Interfacial Assembly. **a** Schematic representation of deposition of MoS_2_ films from water-hexane interface. **b** AFM scan of 20 × 20 μm^2^ film area.

In a 500 mL beaker with a PTFE magnetic stirrer, ~ 300 mL of deionised water (>18M*Ω*cm) and 50 mL n-hexane (≥99%, Sigma Aldrich) were added. Figure 5. A homebuilt PTFE substrate stage was loaded with the required substrates and submerged beneath the water-hexane interface. The MoS_2_ ink was added to a 5 mL syringe (2-part, NORM-JECT, VWR) and loaded into a syringe pump (Model: SPM, DK Infusetek, Shanghai). The stirring was started at 100 RPM (Micro Stirrer F203A0440, Kleinfield, Germany), and MoS_2_ was added to the interface at a rate of 0.15 mL min^−1^. Once a complete film was formed at the interface, stirring was stopped and the substrates were lifted at a rate of 1 mm s^−1^ (Dip Coater, Ossilla, Netherlands) (Fig. 5a). After deposition, the substrates were air dried before being dried under vacuum at 50 °C. Network of electrochemically exfoliated monolayer MoS_2_ nano-sheets covers uniformly surface with average 80–90% coverage (Fig. 5b).

**Fig. 6:**
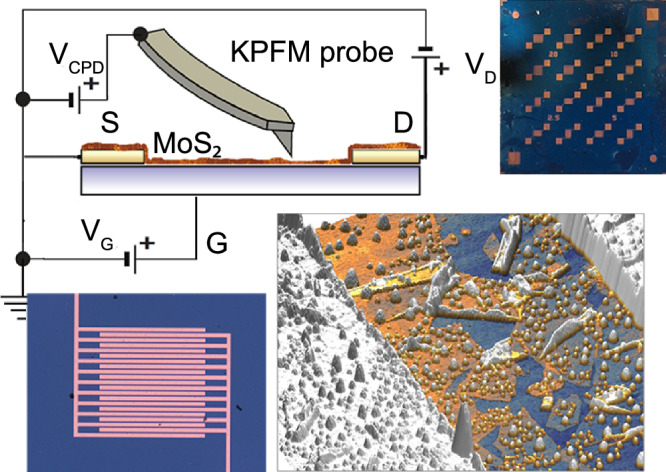
Schematic representation of KPFM measurement configuration (not to scale) for in-operando MoS_2_ FET analysis. The lower right section provides a three-dimensional view of the FET channel. Inset: Photo of the TFT MoS_2_ with 5 μm channel.

MoS_2_ TFTs on silicon-based OFET substrates (Fraunhofer, Germany) with 90nm SiO_2_ oxide and pre-patterned gold contacts Fig. 6. All transistors have *W* = 10 mm and we analysed two different channel length, L = 2.5, and 5 μm. Figure 6 inset.

The residue present at surface and captured in overlaps consist mainly of polyvinylpyrrolidone (PVP) from the ink, together with trace organics, solvent and adsorbed water. Although repeated IPA rinses (with N_2_ drying) greatly lessen this contamination, a thin layer of PVP/organics usually persists. Residual layer act as a nm-scale spacer/dielectric, increasing the effective tunnelling/hopping barrier and giving the characteristics of the type 2 junctions. Achieving a highly connected MoS_2_ network requires removing residue and encouraging extended, void-free van-der-Waals contact between neighbouring flakes while minimising twist. This could be perused by few possible techniques. By formulating low-residue inks–reducing the PVP content or replacing it with shorter, more volatile ligands, or by performing a ligand exchange to species that can be readily removed–and by subjecting the deposited films to a mild thermal anneal (150–250 °C in Ar or forming gas) to desorb solvent and soften any remaining organics without damaging the nanosheets. Additional gains can be realized by tuning the substrate surface energy: a mild O_2_-plasma clean followed by a hexamethyldisilazane (HMDS) prime suppresses interfacial water and sets a contact angle that favours spreading and close contact^44,45^ Finally, gentle mechanical compression, such as light lamination or pressing after partial drying, could further improve conformity at the overlaps and thus reduce junction resistance.

### AFM and KPFM

AFM and FM-KPFM measurements were performed using a Horiba/AIST-NT Omegascope AFM system. NuNano SPARK 350 Pt probes were used with a spring constant of 42 Nm^−1^, resonant frequency 350 kHz, and tip radius of 30 nm (Fig. 6) FM-KPFM measurements were carried out in a two-pass mode, with the probe lifted by 15 nm in the second pass (Fig. 6). Extraction of the potential drop maps from KPFM is presented at SI and 1.2 and Fig. S4. Topography and CPD images were processed in the open-source software Gwyddion v2.56.

### Raman spectroscopy and Photoluminescence

Raman measurements were performed using a Horiba LabRam HR Evolution confocal Raman spectrometer using 1800 lines/mm gratings. A 532 nm laser source was used to excite the samples with an excitation power of 0.1-3.2 mW. The laser spot was focused by a 100x, 0.9 NA objective.

## Supplementary information

Supplementary Information

## Data Availability

Data is provided within the manuscript and supplementary information files. Raw data are available from the authors on reasonable request.
