# Supplementary material for: Imaging junctions in two-dimensional semiconductor nanosheet networks
Source: NPJ 2D Mater Appl. 2025 Oct 31;9(1):90. doi: 10.1038/s41699-025-00609-6 (PMC12578641; doi:10.1038/s41699-025-00609-6)
Supplement: Supplementary file 1 — Supplementary Information [file 41699_2025_609_MOESM1_ESM.pdf]

Imaging Junctions in Two-dimensional Semiconductor Nanosheet Networks

Jelena Pešić[1,2*†], Simon Leitner[1†], Joseph Neilson[3], Igor Stanković[1,4], Muhammad Zubair Khan[1], Dragana Tizić Matković[5], Adam G. Kelly[3,6], Tian Carey[3], Jonathan Coleman[3], Aleksandar Matković[1]

[1*]Chair of Physics, Department Physics, Mechanics and Electrical Engineering, Montanuniversität Leoben, Franz Josef Strasse 18, 8700 Leoben, Austria.
[2]Laboratory for 2D Materials, Center for Solid State Physics and New Materials, Institute of Physics Belgrade, University of Belgrade, Pregrevica 118, 11080 Belgrade, Serbia.
[3]School of Physics, CRANN & AMBER Research Centres, Trinity College Dublin, Dublin 2, Ireland.
[4]Scientific Computing Laboratory, Center for the Study of Complex Systems, Institute of Physics Belgrade, University of Belgrade, Pregrevica 118, 11080 Belgrade, Serbia.
[5]Chair of Resource Mineralogy, Montanuniversität Leoben, Peter Tunner Strasse 5, 8700 Leoben, Austria.
[6]I3N/CENIMAT, Faculty of Science and Technology, Universidade NOVA de Lisboa, Campus de Caparica, 2829-516, Caparica, Portugal.

*Corresponding author(s). E-mail(s): jelena.pesic@ipb.ac.rs;
Contributing authors: COLEMAJ@tcd.ie; aleksandar.matkovic@unileoben.ac.at;
[†]These authors contributed equally to this work.

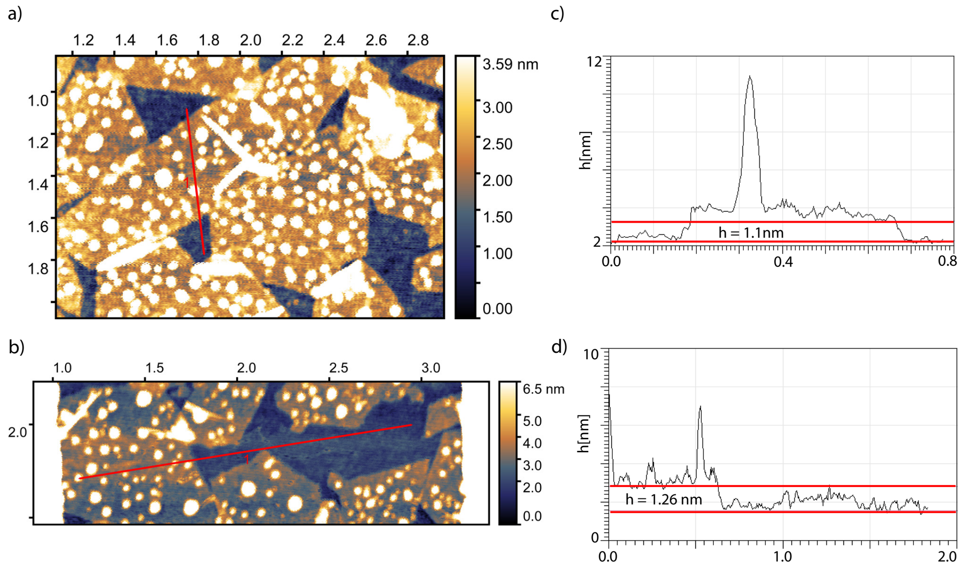

Fig. S1 (a, b) Representative AFM topography images of isolated nanosheets deposited on SiO_2/Si (colour scale in nm). (c, d) Height profiles extracted along the dashed lines in (a) and (b) give step heights of 1.1 nm and 1.26 nm, respectively, verifying that the flakes are single layers; the slightly larger values than the ideal 0.7 nm lattice spacing are typical for solution-processed MoS_2 on oxide substrates and arise from adsorbed water or residual stabiliser.

1 Extended Data

1.1 Validation of Monolayer MoS_2 flakes

AFM topography with line profiles (Fig. S1) shows step heights, matching our earlier report [1, 2], widely observed for liquid-phase-exfoliated monolayer MoS_2 on SiO_2, where adsorbed water and residual stabiliser layers raise the apparent thickness above the crystallographic 0.65–0.7 nm value. The uniform colour contrast across all flakes in the AFM topography maps indicates that they have the same thickness. AFM line profiles indicate apparent heights of 1–1.2 nm—typical for liquid-phase-exfoliated monolayers on SiO_2 owing to adsorbed water and residual stabiliser. These residues, evident as thin patches in the topography, can act as nm-scale dielectric spacers at overlaps and contribute to the junction resistance. Thickness of the single flake is confirmed by Raman spectroscopy. We estimate that more than 95% of the flakes are monolayers.

Using Raman spectroscopy(Fig. S2a), we confirmed that most of the samples are monolayer MoS_2 flakes. Raman spectra show the expected E_{2g} - A_{1g} peak separation of approximately 18 cm^{-1}. We also provide Raman and PL spectra of the sample region containing a thicker flake (2L). PL spectra (Fig. S2b) shows clear quenching of emission for the bilayer region. The optical spot of a conventional confocal PL microscope is ∼400 nm, larger than both the average flake radius (∼300 nm)

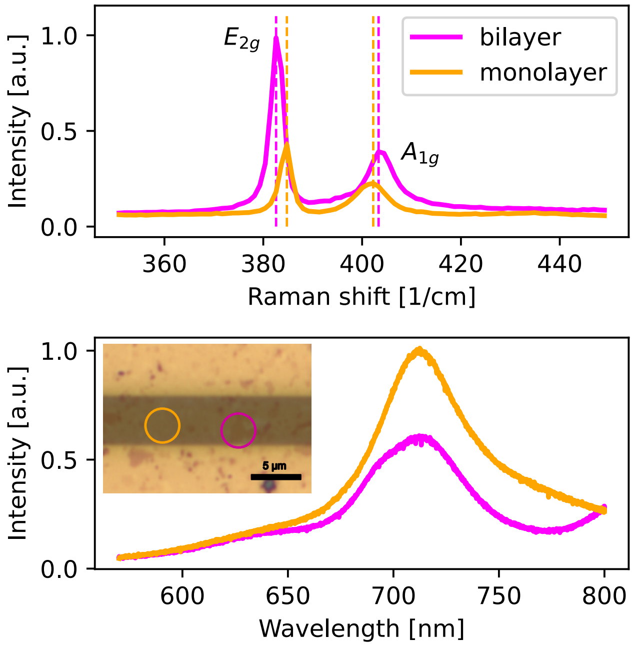

Fig. S2 a) Raman spectra for monolayer (orange) and bilayer (magenta) MoS_2. The dashed lines indicate E_{2g} and A_{1g} modes. The separation between the E_{2g} and A_{1g} modes of about 18 cm^{-1} for the orange curve is a spectral signature of the monolayer. b) PL spectra of the same regions investigated in a). A clear quenching of emission is observed for the bilayer region. The inset shows an optical image of the investigated regions, with circles denoting the measurement spots. Over 95% of the film surface exhibits optical contrast corresponding to monolayers (orange curves)

and most overlap widths, below ~100 nm, standard PL mapping cannot fully resolve individual sheets from their junctions in device channels. Attaining the necessary sub-100 nm optical resolution remains an open challenge and could be addressed in future work by tip-enhanced PL (or Raman) mapping, which would enable direct, nanoscale correlation of local optical signatures with the electrostatic potential landscape revealed by KPFM. Scanning-electron microscopy image (Figure S3) of the deposited nanosheet film on SiO_2/Si, showing a densely interconnected network of predominantly monolayer flakes (typical lateral size $\sim 0.3\,\mu m$) with partial overlaps that form the conduction paths analysed in the main text.

1.2 AFM and FM-KPFM measurements and analysis

The following procedure was followed to obtain voltage drop maps and cross-sections from KPFM experiments. In the presented example (Fig S4), the scanned areas included dimensions of $1.5 \times 4\,\mu m^2$. The analysis line for potential drop assessment is denoted by the gray marker. When all electrodes are grounded, the CPD map (A) reveals both work function differences and topography-related artifacts, which are used for the normalization process. In the same measured area with an applied bias of

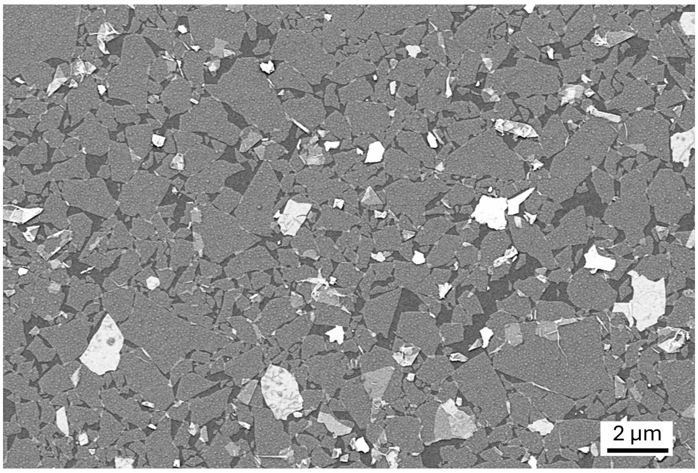

Fig. S3 SEM image showing MoS_2 LPE nanosheet film on SiO_2/Si, showing a densely interconnected network of predominantly monolayer flakes with partial overlaps that form the conduction paths analyzed in the main text. The scale bar corresponds to 2 µm

+2 V, the CPD map (B) captures both work function variances and potential drops. Subtracting the 0-bias map from the map during operation (A-B) effectively eliminates most potential drop sources, leaving only the voltage drops associated with FET operation.

1.3 Junction analysis

We identify three typical examples of junctions that can form between two sheets. In Fig S5, the topography of each type of junction is presented, with shading in color coding that matches the main text.

The resolution of AFM can be limited by several factors when measuring very small features. The finite radius of the AFM tip can affect the resolution, as a tip larger than the feature may not accurately resolve small overlaps or fine structures. Additionally, the inherent resolution of AFM is constrained by the precision of the feedback mechanisms and the signal-to-noise ratio, which can make it challenging to distinguish between overlapping structures. Moreover, interactions between the AFM tip and the surface can lead to distortion or blurring, further complicating the precise definition of extremely small features. Hence all features we notice in CPD maps but we can not resolve in topography we classify them as 9.999 nm to maintain a consistent comparison.

We examined potential profiles specifically for regions where continuous MoS_2 flakes bridge the electrode edges, enabling an unambiguous attribution of the local voltage step to the contact resistance (Figure S6). This analysis yields an average contact-related drop of 10±2%. Furthermore, the voltage drop is localized almost entirely at the grounded (source) electrode, consistent with an electron-injection barrier characteristic of a Schottky-type Au/MoS_2 contact.

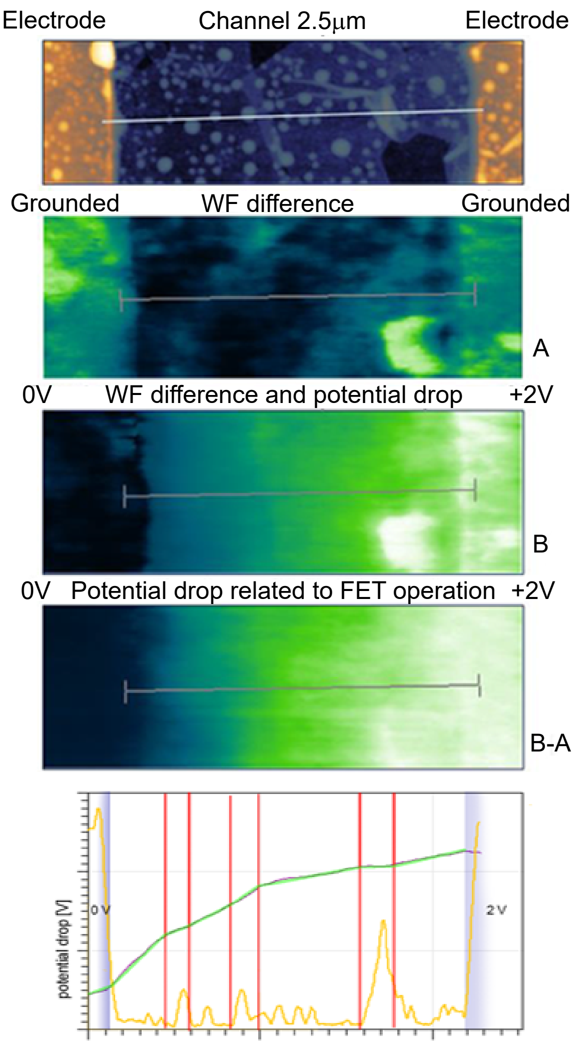

Fig. S4 Extraction of the potential drop maps from KPFM experiments. Bottom panel: Yellow line represents topography on the selected current path and green is resulting potential drop for operational FET. Red lines are guides to eye for junctions on current path.

1.4 Electrical characterization

Electrical characterization was done using a Keithley 2636A Source-Meter attached to an Instec probe station. The samples were contacted with Au coated Ti electrical cantilever microprobes. The temperature was controlled with liquid nitrogen and an electrical heating stage, and was monitored via a mK2000 temperature controller connected to the probe station with a temperature resolution of 0.01 K.

On-off ratios $I_{on/off}$ were found to be between 10^3 and 10^4, with one exemplary transfer curve shown in Fig S7. Figure S8 illustrates the Schottky-barrier-height (SBH)

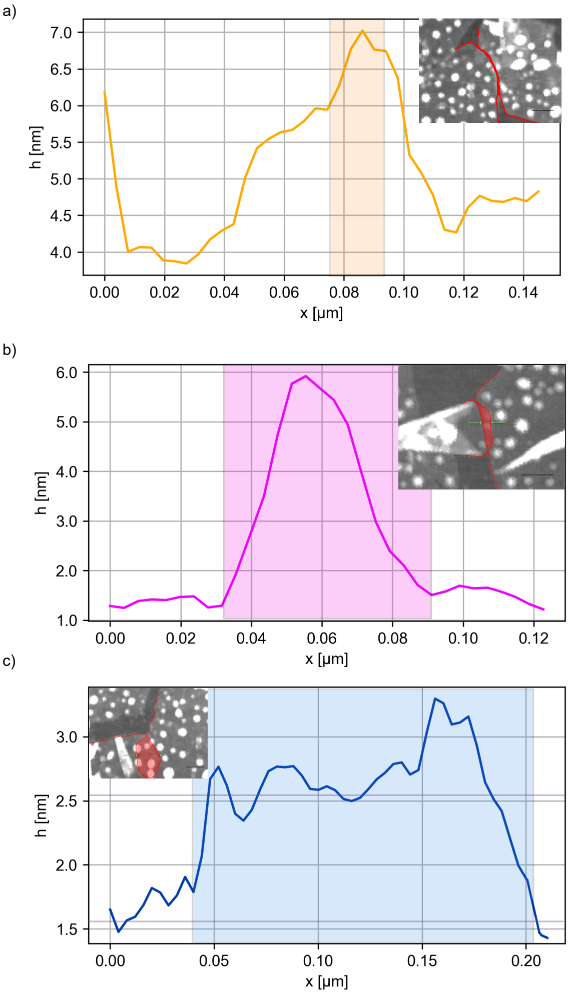

Fig. S5 Topography profile for three types of junctions.

analysis for a 5 µm-channel device. For each gate bias, we construct an Arrhenius plot of the drain current, I_D, versus inverse temperature; the slope of the linear region in these plots (inset) yields the apparent SBH at that bias. The effective SBH is taken at the flat-band voltage—identified as the point where the data deviate from the gate-voltage-dependent linear trend, marked by the red dashed lines. We note that the extracted barrier height represents the combined influence of the metal–semiconductor contact and the distributed N-NS junction resistances within the channel. Figure S9 shows the output characteristics of the device; a distinct S-shaped curvature appears in the low V_{DS} regime, deviating from the ideal linear behaviour expected for purely ohmic contacts. This "S-bending" arises when the contact and inter-flake junction resistances are comparable to, or larger than, the intrinsic channel resistance, producing an additional voltage drop that must be overcome before appreciable current can

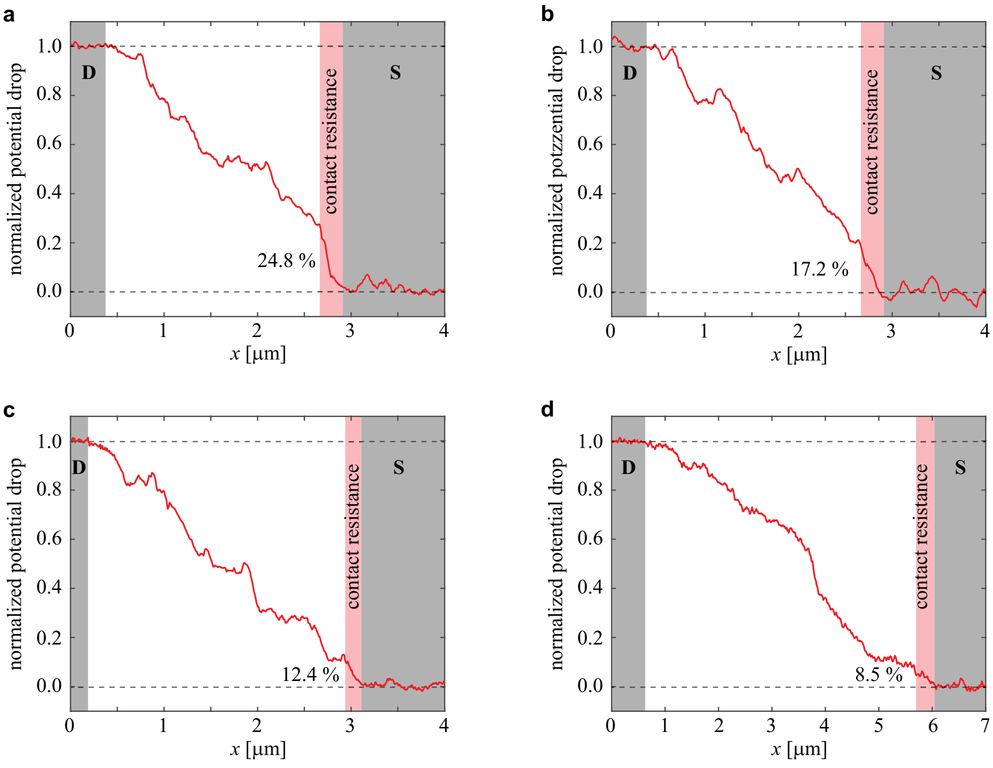

Fig. S6 (a-d) examples of the normalized potential drop profiles where the nanosheets cross over the contacts and where the potential drops associated with the contact resistance are clearly identified. Shaded gray areas indicate source (S) and drain (D) regions, and red shaded regions indicate the potential drops associated with the contact resistance. (a) and (b) show some of the highest observed contact contributions, while (c) and (d) show representative contact contributions. Considering all the potential drop profiles where the contact resistance can be clearly separated, a contact contribution of (10 ± 2) % was estimated.

flow. The feature therefore serves as a qualitative indicator of the high series resistance discussed in the main text.

1.5 Y function-based device modelling

Modelling of the sets of the electrical transfer curves was carried out based on the procedure described in [3]. The device is modelled as an ideal long-channel transistor operating in the linear regime connected to the drain bias in a series with a resistor that sums up all the junction and contact resistances in the system. The drain-source current can be expressed using the equation:

$$I_D = \frac{\mu_0 C_i}{1 + \theta \times \left(V_G - V_{th} - \frac{V_D}{2}\right)} \frac{W}{L} \left(V_G - V_{th} - \frac{V_D}{2}\right) V_D, \tag{1}$$

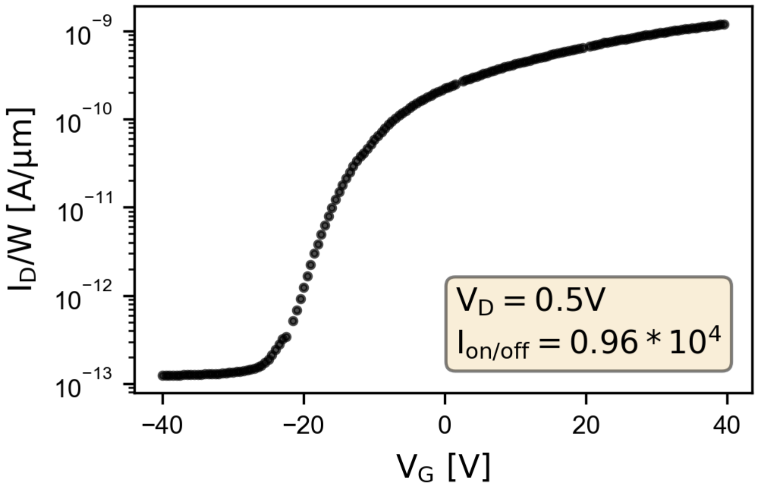

Fig. S7 Transfer curve in semi-log scale showing an on-off ratio of $0.96 * 10^4$.

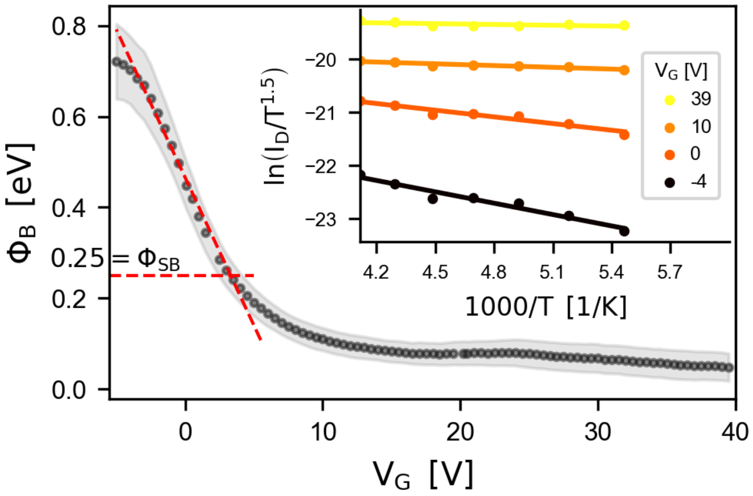

Fig. S8 Schottky barrier height extraction for a 5 µm channel device. An Arrhenius plot based Schottky barrier height (SBH) fitting procedure is performed, where the slope of the curves shown in the inset corresponds to the Schottky barrier height at a given gate bias. This slope is then plotted against the corresponding gate voltage in the main figure. As the Schottky barrier changes with different gating, the effective Schottky barrier height is extracted at the flatband voltage, which is indicated as the point at which the data leaves a linear fit, shown by red dashed lines. Grey contours to the data indicate a one-sigma confidence band obtained from the goodness of the linear fit in the inset. Further, it is important to mention that both contact and NS-NS junction resistances are contributing to the SBH estimation.

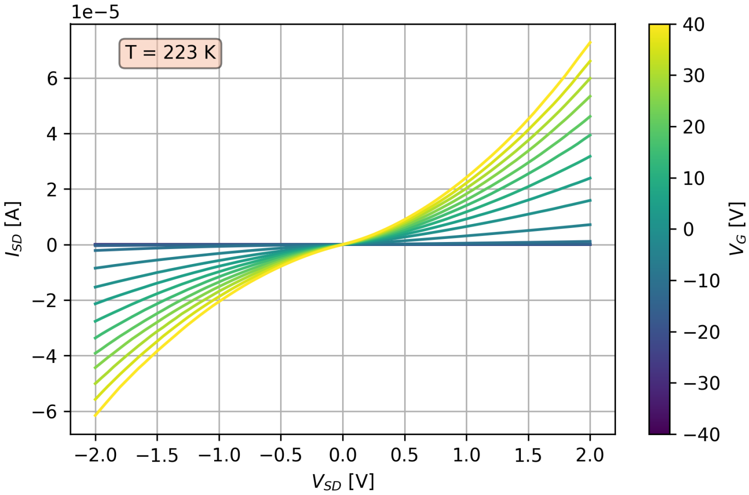

Fig. S9 Electrical output curves (5 um channel length, measured at 223 K) measured at varied gate bias starting from an on-state at +40 V (yellow curve) and reducing the gate bias in the steps of 5 V. A clear non-linearity in the low drain-source bias region is a signature of a significant junction and contact resistance contributions. Please mind that the contributions from the NS-NS junctions and the contact resistances both contribute to the non-linear output at low bias.

where: $\theta = 2R_C\mu_0 C_i \frac{W}{L}$. The model yields three fitting parameters: μ_0, V_{th}, and R_C. The Y function model, fits the $Y \equiv \frac{I_D}{\sqrt{g_m}}$ and $\frac{1}{\sqrt{g_m}}$ as functions of V_{G} to determine the slopes, defined as S_1 and S_2 parameters, respectively.

1.6 The model

In our study, we employed Voronoi diagrams to model and analyze a network of conductive flakes. Voronoi diagrams are a well-established mathematical method for partitioning a plane into regions based on the distance to a specific set of points. These regions, called Voronoi cells, are constructed such that any point within a given cell is closer to its generating point (seed) than to any other seed. This method is particularly advantageous for modeling heterogeneous materials and networks due to its ability to represent irregular and randomly distributed structures.

To generate our network of conductive flakes, we followed a systematic approach. Initially, seeds were randomly distributed within a defined plane, with each seed acting as the center of a Voronoi cell. Using these seeds, Voronoi cells were constructed, where each cell represented an individual flake within the conductive network. The edges of these cells denote potential conductive paths between neighboring flakes. The probability that an edge between two touching flakes is non-conductive is assumed to be proportional to the inverse square of the edge's length. This assumption is grounded in the idea that longer edges have a higher likelihood of forming conductive bridges due to a greater contact area.

The electrical conductivity of the flakes and their connecting edges is a critical factor in our model. Each flake is assumed to be 10 times more conductive than the edges,

reflecting the physical reality that within-flake conduction is typically much more efficient than conduction across the junctions between flakes. As already explained, the connectivity between flakes is probabilistic and depends on its length squared, capturing the variability and randomness inherent in real-world conductive networks.

To evaluate the electrical properties of the network, we applied Kirchhoff's laws to the network of pixels representing the conductive flakes and their connections. The system is discretized and the conductivity is assigned to each pixel, depending if it is part of the flake, conductive, or non-conductive edge. Kirchhoff's Current Law (KCL) is applied to ensure that the sum of currents entering and leaving any node is zero.

To simulate real-world applications, we placed electrodes on the top and bottom boundaries of the network. This setup creates a potential difference across the network, driving current through the conductive paths formed by the flakes and their edges. The boundary conditions for the simulation were established by setting the top electrode to a constant voltage (V_{top}) and the bottom electrode to ground potential ($V_{bottom} = 0$). These boundary conditions allow us to study the macroscopic conductive properties of the network, such as total resistance and current distribution.

The aspect ratio and size of the flakes are free parameters in our model. By varying these parameters, we can explore their impact on the overall conductivity of the network. Different aspect ratios and flake sizes result in different Voronoi tessellations, influencing the connectivity and the probability of edge conduction. In summary, our approach leverages the Voronoi diagram's capacity to model complex, random networks of conductive flakes. By incorporating probabilistic edge conductivity and applying Kirchhoff's laws, we can accurately simulate and analyze the electrical properties of these networks, providing valuable insights into the design and optimization of conductive materials.

We matched geometry parameters, length-width ratio and number of seeds, and conductivity parameters, percolation factor (probability for the edge to be non conductive) and edge-to-island conductivity ratio.

1.7 Model Validation, Channel Width and Length Analysis

To align the conductivity parameters with experimental data from KPFM measurements, we adjusted the percolation factor and conductivity ratio. If the probability of conduction between nanosheets were doubled, more frequent and less defined current paths would form between the electrodes. However, if the system had half the probability of conduction, the current paths would form less frequently.

With parameters matched to experimental results, we tested our model on larger channel widths and lengths. We still observed that approximately 25% of the total device channel width contributes to the current flow from source to drain.

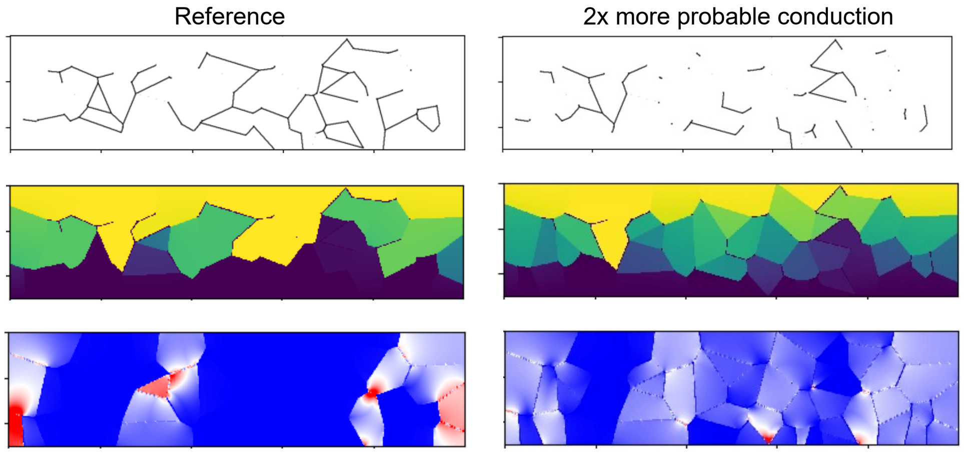

Fig. S10 Impact of doubling the conduction probability between nanosheets. Referent and tested model are scaled on $3 \times 15\,\mathrm{\mu m}^2$

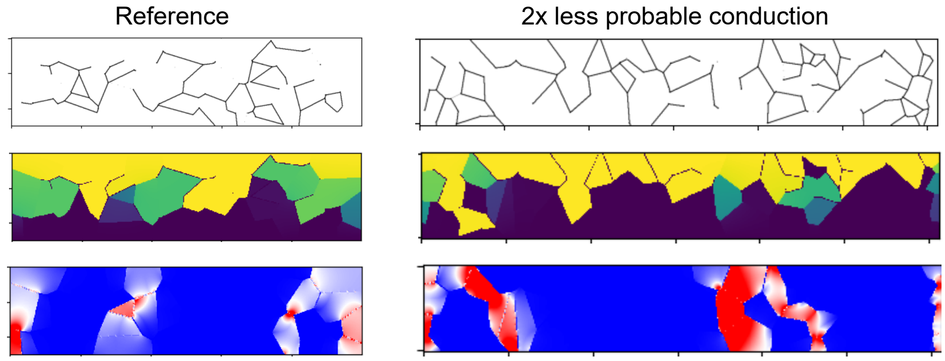

Fig. S11 Impact of halving the conduction probability between nanosheets. This results in less frequent but more spatially defined current paths forming between the electrodes. Tested model is scaled on $3 \times 21\,\mathrm{\mu m}^2$

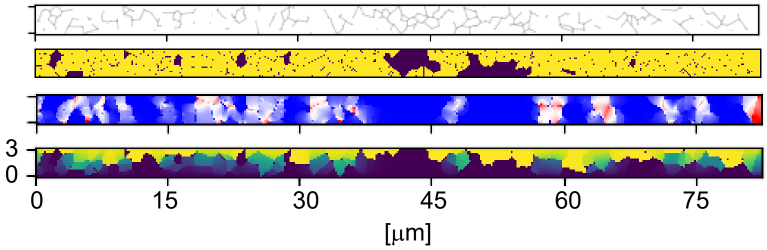

Fig. S12 Model validation for devices with very long channel widths. Approximately 25% of the total channel width contributes to the current flow from source to drain.

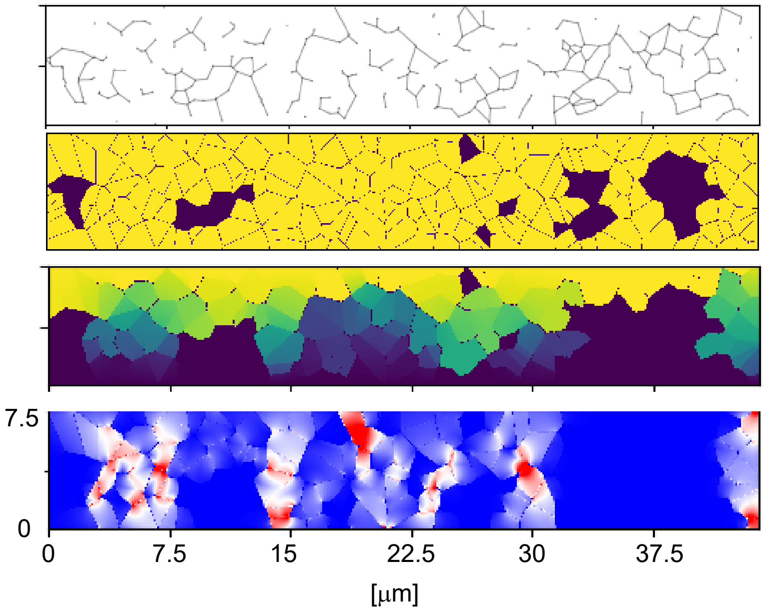

Fig. S13 Model validation for devices with longer channel lengths. The current flow dynamics and distribution across the extended channel length are tested resulting in 22-28% of the total channel width contributes to the current flow

References

[S1] Kelly, A.G., Hallam, T., Backes, C., Harvey, A., Esmaeily, A.S., Godwin, I., Coelho, J., Nicolosi, V., Lauth, J., Kulkarni, A., Kinge, S., Siebbeles, L.D.A., Duesberg, G.S., Coleman, J.N.: All-printed thin-film transistors from networks of liquid-exfoliated nanosheets. Science **356**(6333), 69–73 (2017)

[S2] Neilson, J., Caffrey, E., Cassidy, O., Gabbett, C., Synnatschke, K., Schneider, E., Munuera, J.M., Carey, T., Rimmer, M., Sofer, Z., Maultzsch, J., Haigh, S.J., Coleman, J.N.: Production of ultrathin and high-quality nanosheet networks via layer-by-layer assembly at liquid–liquid interfaces. ACS Nano **18**(47), 32589–32601 (2024) https://doi.org/10.1021/acsnano.4c09745

[S3] Jain, A., Szabo, A., Parzefall, M., Bonvin, E., Taniguchi, T., Watanabe, K., Bharadwaj, P., Luisier, M., Novotny, L.: One-dimensional edge contacts to a monolayer semiconductor. Nano Letters **19**(10), 6914–6923 (2019) https://doi.org/10.1021/acs.nanolett.9b02166
